# Spontaneous pulmonary emphysema in mice lacking all three nitric oxide synthase isoforms

**DOI:** 10.1038/s41598-021-01453-6

**Published:** 2021-11-11

**Authors:** Kaori Kato, Masato Tsutsui, Shingo Noguchi, Yukitoshi Iha, Keisuke Naito, Takaaki Ogoshi, Chinatsu Nishida, Masahiro Tahara, Hirotaka Yamashita, Ke-Yong Wang, Yumiko Toyohira, Nobuyuki Yanagihara, Hiroaki Masuzaki, Hiroaki Shimokawa, Akihide Tanimoto, Kazuhiro Yatera

**Affiliations:** 1 Department of Respiratory Medicine, University of Occupational and Environmental Health, Japan, Kitakyushu, Japan; 2 Department of Pharmacology, Graduate School of Medicine, University of the Ryukyus, 207 Uehara, Nishihara, Okinawa, 903-0215 Japan; 3 Shared-Use Research Center, University of Occupational and Environmental Health, Japan, Kitakyushu, Japan; 4 Department of Pharmacology, School of Medicine, University of Occupational and Environmental Health, Japan, Kitakyushu, Japan; 5 Second Department of Internal Medicine, Graduate School of Medicine, University of the Ryukyus, Okinawa, Japan; 6 Department of Cardiovascular Medicine, Tohoku University Graduate School of Medicine, Sendai, Japan; 7 Department of Pathology, Kagoshima University Graduate School of Medical and Dental Sciences, Kagoshima, Japan

**Keywords:** Diseases, Pathogenesis

## Abstract

The roles of endogenous nitric oxide (NO) derived from the entire NO synthases (NOSs) system have yet to be fully elucidated. We addressed this issue in mice in which all three NOS isoforms were deleted. Under basal conditions, the triple n/i/eNOSs^−/−^ mice displayed significantly longer mean alveolar linear intercept length, increased alveolar destructive index, reduced lung elastic fiber content, lower lung field computed tomographic value, and greater end-expiratory lung volume as compared with wild-type (WT) mice. None of single NOS^−/−^ or double NOSs^−/−^ genotypes showed such features. These findings were observed in the triple n/i/eNOSs^−/−^ mice as early as 4 weeks after birth. Cyclopaedic and quantitative comparisons of mRNA expression levels between the lungs of WT and triple n/i/eNOSs^−/−^ mice by cap analysis of gene expression (CAGE) revealed that mRNA expression levels of three Wnt ligands and ten Wnt/β-catenin signaling components were significantly reduced in the lungs of triple n/i/eNOSs^−/−^ mice. These results provide the first direct evidence that complete disruption of all three NOS genes results in spontaneous pulmonary emphysema in juvenile mice in vivo possibly through down-regulation of the Wnt/β-catenin signaling pathway, demonstrating a novel preventive role of the endogenous NO/NOS system in the occurrence of pulmonary emphysema.

## Introduction

Pulmonary emphysema is a chronic lung condition that causes shortness of breath and is defined as the “abnormal permanent enlargement of alveolar air-space size distal to the terminal bronchioles, accompanied by destruction of the alveolar wall and without obvious fibrosis”. Chronic obstructive pulmonary disease (COPD), which includes pulmonary emphysema, is typically caused by long-term exposure to irritating gases or particulate matter, most often from tobacco smoke. Obstructive ventilatory disturbance due to COPD is usually progressive and sometimes shows exacerbation due to infection, which lowers life expectancy. In cross-sectional epidemiological studies worldwide, the prevalence of COPD in people over 40 years is around 10%^1^, and the World Health Organization (WHO) global health estimates report that COPD is the third leading cause of death worldwide in 2019, with an estimated 3.3 million deaths (6% of the world’s total deaths). The number of COPD patients is expected to continue to increase due to an aging population and continued use of tobacco^2^. Bronchodilators, including anti-cholinergic agents and β_2_-adrenergic receptor stimulants are used in the treatment of symptomatic patients with COPD; however, since the precise pathogenesis of pulmonary emphysema has not been fully clarified, its definitive treatment has not yet been established.

Nitric oxide (NO) plays important roles in maintaining homeostasis of the respiratory system. NO is synthesized from l-arginine by three distinct isoforms of NO synthase (NOS), including neuronal (nNOS), inducible (iNOS), and endothelial NOS (eNOS); all of which are expressed in human lung tissues under both physiological and pathological conditions^3–5^. nNOS is present in the airway epithelium, vascular endothelium, vascular smooth muscle cells (VSMCs), and nerve cells; iNOS is expressed in the airway epithelium, vascular endothelium, VSMCs, and alveolar macrophages; and eNOS is localized in the vascular endothelium, VSMCs, and type II alveolar epithelial cells^6–8^. There has been no previous study that characterizes the role of nNOS in pulmonary emphysema. Previous studies reported that pulmonary emphysema induced by elastase or tobacco smoke is mitigated in iNOS^−/−^ mice to a greater extent than in wild-type (WT)^8,9^, whereas another study showed that pulmonary emphysema induced by elastase is comparable in iNOS^−/−^ and WT mice^10^. Similar extents of elastase-induced pulmonary emphysema in eNOS^−/−^ and WT mice have been indicated^10^.

The in vivo roles of endogenous NO derived from the entire NOS system in pulmonary emphysema have been examined in pharmacological studies with non-selective NOSs inhibitors, such as *N*^*ω*^-nitro-l-arginine methyl ester (l-NAME). It has been reported that long-term treatment with l-NAME alleviates tobacco smoke-induced pulmonary emphysema in mice^11^; however, it has also been shown that long-term treatment with the NO precursor l-arginine also reduces pulmonary emphysema, suggesting that the above-mentioned long-term effects of l-NAME are not caused primarily by the inhibition of NOSs^11^. The following lines of evidence support this notion: first, long-term vascular effects of l-NAME also are not caused by the inhibition of NOSs^12^; second, long-term treatment with l-NAME does not reduce eNOS activity^13^; third, multiple actions of l-NAME other than simple inhibition of NOSs have been reported^14,15^. Thus, due to lack of specificity of this inhibitor, the authentic roles of NOSs in the pathogenesis of pulmonary emphysema remains unclear. The most appropriate way to address this issue is to use mice that are deficient in the entire NOS system (i.e. triple n/i/eNOSs^−/−^ mice)^16^. Here, we demonstrate that the triple n/i/eNOSs^−/−^ mice spontaneously developed pulmonary emphysema.

## Methods

### Animals

This study was approved by the Ethics Committee of Animal Care and Experimentation, the University of Occupational and Environmental Health, Kitakyushu, Japan. The study was carried out in compliance with the ARRIVE guidelines and in accordance with the American Veterinary Medical Association (AVMA) Guidelines. Experiments were performed in male wild-type (WT) C57BL/6J (Charles River, Yokohama, Japan), single nNOS^−/−^, iNOS^−/−^, eNOS^−/−^, double n/iNOSs^−/−^, n/eNOSs^−/−^, i/eNOSs^−/−^, and triple n/i/eNOSs^−/−^ mice^16^. The mice were maintained on a regular diet (CE-2, CLEA Japan, Inc., Tokyo, Japan). Sample size was empirically chosen. There was no exclusions of animals or data points.

### Plasma nitrite plus nitrate (NOx) levels

Blood samples were taken from the right axillary artery at the time of sacrifice and were collected using vacuum tubes containing sodium EDTA. The blood samples were immediately centrifuged at 3000 rpm at 4 °C for 15 min, and the supernatant plasma was stored at − 80 °C. Plasma NOx concentrations were measured by the Griess method using the ENO-20 NOx analysis system (Eicom, Kyoto, Japan), as previously described^16^.

### Histopathology

Mice were sacrificed at 8 weeks of age and the lungs were fixed with 15% formalin neutral buffer solution (Wako, Osaka, Japan) at 25 cmH_2_O. The tissues were embedded in paraffin and 3-μm-thick sections of the embedded lung tissues were stained with an hematoxylin and eosin solution or an elastic van Gieson (EVG) solution. Mean alveolar linear intercept length was determined by light microscopy at a total magnification of 100×, and 15 random microscopic images per lung tissue section were assessed by microscopic projection onto a reference grid, as previously reported^10^. The mean alveolar linear intercept length was calculated by dividing the total grid length by the number of intersections of alveolar wall gridlines. The alveolar destructive index was analyzed on five randomly selected frames per lung at 200× power, and was determined by means of 42-point grid as previously reported^17^. Structures lying under these points were classified as destroyed alveolar and/or duct space. Alveolar spaces were classed as destroyed if the wall of the alveolus was disrupted in 2 or more places or there were 2 or more disruptions of contiguous alveoli that were part of the structures opening onto a single duct system. Duct spaces were classed as destroyed if 2 or more isolated islands of lung parenchyma were observed in the lumen of a duct. Clearly abnormal morphology or classic emphysematous change were classified as destroyed, too. The alveolar destructive index was calculated as [D/D + N × 100]: D = number of destroyed points, N = number of normal points. EVG staining is useful for identifying the elastic fibers, and the content of the elastic fibers was estimated by measuring the lengths of EVG staining-positive elastic fibers (black color) in random 10 fields per lung tissue with a light microscope at a magnification of 400×, and the average values were compared.

### Microscopic computed tomography (micro CT)

Micro CT analysis was performed under general anesthesia with sevoflurane inhalation (Pfizer Japan, Tokyo, Japan) using a micro CT system (CosmoScan GX, Rigaku Co., Tokyo, Japan). Operating conditions of micro CT were as follows: 90 kV, 88 μA; chest CT: respiratory reconstruction mode; field of view: 25 mm; voxel size: 60 × 60 × 60 μm; and scan time: 4.0 min. Micro CT images of end-expiratory lung volumes and lung field CT values were calculated using an analyze 12.0 software (Analyze Diet, Kansas, USA). Lung parenchyma was defined as a region with X-ray attenuation values between − 1200 and − 300 HU (Hounsfield Unit) according to a previous study^18^, and intrapulmonary and surrounding extrapulmonary tissues (e.g., airways, large pulmonary vessels, heart, mediastinal structures, and diaphragm) were automatically excluded^18^.

### Bronchoalveolar lavage fluid (BALF)

Bronchoalveolar lavage was carried out by cannulating the trachea with a 20-gauge catheter. The lung was lavaged with three aliquots of 1.5 ml saline (0.9% NaCl). BALF was centrifuged at 700×*g* for 10 min at 4 °C and the supernatants were stored at − 80 °C. The cell pellet was diluted in PBS, and total cell number was counted with a hemocytometer after staining with Turk’s stain solution (Merck, Tokyo, Japan). Differential cell counts were determined using cell suspensions displayed on glass slides with a cytocentrifuge (Cytospin 4, Termo, Kanagawa, Japan). The cells on the glass slides were dried, fixed and stained by the Diff-Quick method (Sysmex, Hyogo, Japan), and three hundred cells were identified under a microscope, as previously described^19^.

### Cap analysis gene expression (CAGE)

CAGE library preparation, sequencing, mapping and gene expression analysis were performed by DNAFORM (Kanagawa, Japan). Briefly, quality and quantity of total RNA were checked by a bioanalyzer (Agilent, CA, U.S.A.) to ensure that the RNA integrity numbers (RINs) were above 7.0 and the A260/280 and A260/230 ratios were above 1.7. First-strand cDNAs were reverse-transcribed from the RNAs, then second-stranded cDNAs were synthesized and selected by the Cap trapping method. After “bar code” tags were attached, CAGE libraries were constructed. Multiplexed 8 CAGE libraries were sequenced using single end reads of 75nt on an Illumina NextSeq 500 sequencer. The sequenced CAGE reads were mapped to the mouse mm9 genome using the BWA software program (version 0.7.12-r1039). The unmapped reads were then mapped by HISAT2 (version 2.0.5). The 5ʹ coordinates of CAGE-tags were input for RECLU clustering, with a maximum irreproducible discovery rate (IDR) was 0.1 and minimum count per million (CPM) value was 0.1. The R package edgeR in the RECLU pipeline was used to perform the differential analysis of the genes^20^. To study the molecular mechanisms for spontaneous pulmonary emphysema in triple n/i/eNOSs^−/−^ mice, gene ontology term enrichment analysis and Kyoto Encyclopedia of Genes and Genomes (KEGG) pathway analysis^21^ were performed using the Database for Annotation, Visualization and Integrated Discovery (DAVID) (https://david.ncifcrf.gov/). Differences with *P* < 0.05 were regarded as statistically significant. The datasets of mRNA sequences were deposited in NCBI's Gene Expression Omnibus (GEO, http://www.ncbi.nlm.nih.gov/geo/) and are accessible through GEO series accession number GSE64521.

### Enzyme-linked immunosorbent assay (ELISA)

Protein expression levels of glycogen synthase kinase (GSK)-3β and lymphoid enhancer-binding factor 1 (LEF1) in the lung were measured by ELISA kits (LSBio, Seattle, WA and Aviva Systems Biology, San Diego, CA, respectively).

### Statistical analysis

Results are expressed as mean ± SEM. For comparison of two groups, a Student’s t-test was performed. For comparison of more than two groups, one-way analysis of variance (ANOVA) followed by Dunnett’s test for multiple comparisons was carried out using the SPSS software. Statistical analysis of the CAGE sequencing data was performed by Danaform Inc. (Kanagawa, Japan) as follows. The data of mRNA expression levels (gene counts) were corrected by size factors in order to normalize them by the normalization method of trimmed mean of M values (TMM). The corrected data were analyzed by the likelihood ratio test on a negative binomial distribution using edgeR as previously reported^22^. The Database for Annotation, Visualization and Integrated Discovery (DAVID) (version 6.8) was used. A value of *P* < 0.05 was considered to be statistically significant.

## Results

### Plasma NOx levels in single, double, and triple NOSs^−/−^ mice

**Figure 1:**
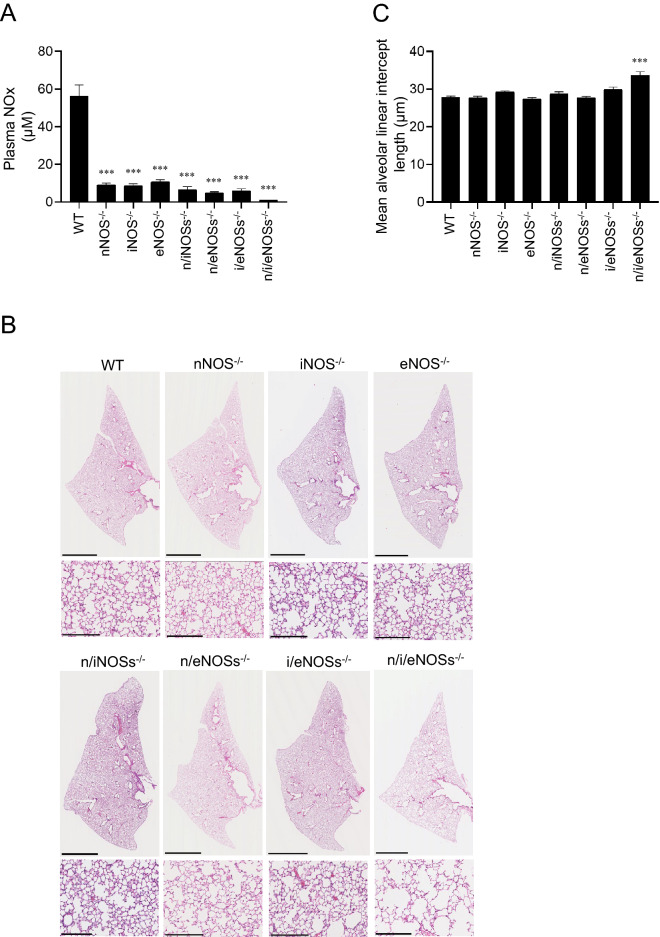

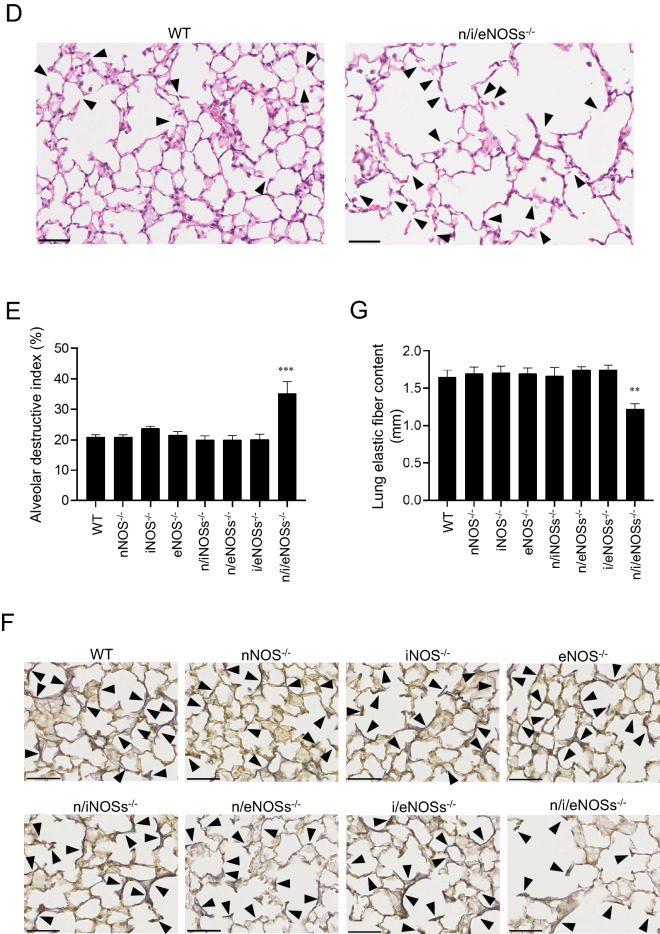
Plasma NOx levels and histopathological examination of the lungs. Experiments were performed in 8-week-old male WT and single, double, and triple NOSs^−/−^ mice. **(A)** Plasma NOx levels. ***P < 0.001 vs. WT mice (n = 9–10). **(B)** Hematoxylin and eosin staining of lung tissues. Scale bars in upper and lower pictures indicate 2,500 and 250 μm, respectively. **(C)** The mean alveolar linear intercept length (n = 9–10). ***P < 0.001 vs. WT mice. **(D)** Hematoxylin and eosin staining of lung tissues in WT and triple n/i/eNOSs^−/−^ mice. Arrowheads indicates destroyed alveoli. Scale bar = 50 μm. **(E)** The alveolar destructive index (n = 5 each) ***P < 0.001 vs. WT mice. **(F)** Elastic van Gieson (EVG) staining of lung tissues. Arrowheads indicate EVG staining-positive elastic fibers (black color). Scale bars represents 50 μm. **(G)** Lung elastic fiber content as estimated by the lengths of EVG staining-positive elastic fibers (n = 4 each). **P < 0.01 vs. WT mice.

In order to evaluate systemic NO production in mice, we measured plasma NOx concentrations in 8 genotypes, including WT, single nNOS^−/−^, iNOS^−/−^, eNOS^−/−^, double n/iNOSs^−/−^, n/eNOSs^−/−^, i/eNOSs^−/−^, and triple n/i/eNOSs^−/−^ mice at 8 weeks of age. The plasma NOx levels were significantly reduced in accordance with the number of disrupted NOS genes in a stepwise manner (Fig. 1A). In the triple n/i/eNOSs^−/−^ mice, the plasma NOx levels were extremely low, with only 1.9% of normal plasma NOx levels in the WT mice (Fig. 1A). These results were in agreement with our previous study^16^.

### Enlarged alveolar air-space size, increased alveolar destruction, and reduced Lung elastic fiber content in triple n/i/eNOSs^−/−^ mice

We performed morphological analysis of 8-week-old mouse lungs stained by hematoxylin and eosin. As compared with the WT mice, neither apparent lung morphological abnormalities nor significant changes of the mean alveolar linear intercept length or the alveolar destructive index, useful parameters for evaluating pulmonary emphysema^23^, were seen in any of the single NOS^−/−^ or double NOSs^−/−^ mice, whereas enlargement of the alveolar air-space size and significant increases in the mean alveolar linear intercept length (Fig. 1B,C) and the alveolar destructive index (Fig. 1D,E) were noted in the triple n/i/eNOSs^−/−^ mice. The lung elastic fiber content, as estimated by the lengths of EVG staining-positive elastic fibers (black color, arrowheads in Fig. 1F,G) was also significantly reduced only in the triple n/i/eNOSs^−/−^ mice compared with the WT mice.

### Reduced lung field CT value and larger end-expiratory lung volume in triple n/i/eNOSs^−/−^ mice

**Figure 2:**
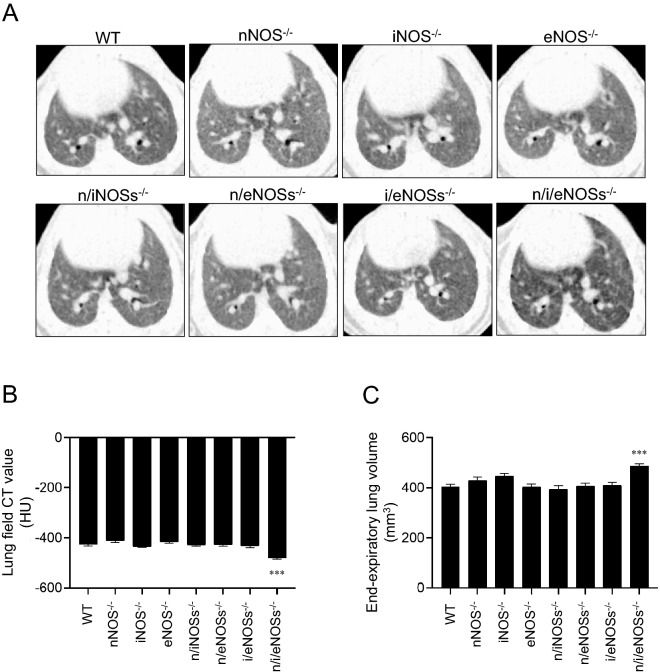
Micro CT analysis of the lungs. Experiments were performed in 8-week-old male WT and single, double, and triple NOSs^−/−^ mice (n = 9–10). **(A)** Micro CT images. **(B)** CT value in the lung field. **(C)** End-expiratory lung volume estimated by micro CT images. ***P < 0.001 vs. WT mice.

We next carried out micro CT analysis in 8-week-old mice. The CT value in the lung field was significantly lower and end-expiratory lung volume was significantly larger in the triple n/i/eNOSs^−/−^ mice, but not in any single NOS^−/−^ or double NOSs^−/−^ mice, as compared with the WT mice (Fig. 2A–C). Taken together with the results of the histopathological analysis, it was conceivable that the triple n/i/eNOSs^−/−^ mice exhibited spontaneous pulmonary emphysema.

**Figure 3:**
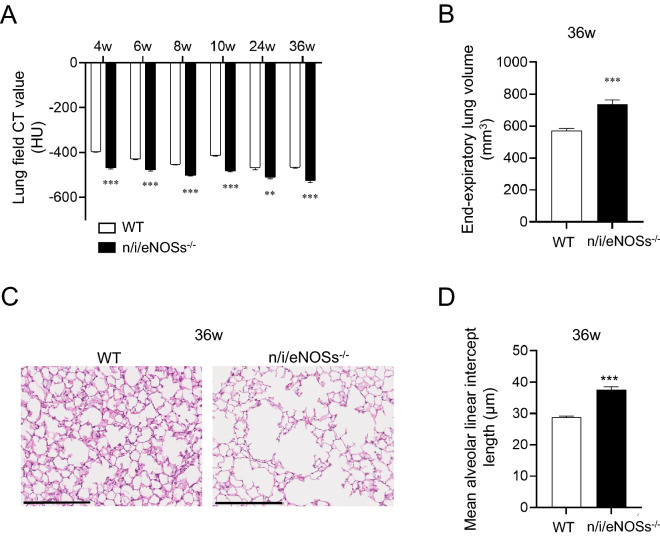
Progression of spontaneous pulmonary emphysema in triple n/i/eNOSs^−/−^ mice. **(A)** Time course of CT value in the lung field in WT and triple n/i/eNOSs^−/−^ mice from 4 to 36 weeks after birth (n = 6–10). **P < 0.01, ***P < 0.001 between WT and triple n/i/eNOSs^−/−^ mice at each time point. **(B)** End-expiratory lung volume at 36 weeks after birth. ***P < 0.001. **(C)** Hematoxylin and eosin staining of lung tissues in WT and triple n/i/eNOSs^−/−^ mice at 36 weeks after birth. Scale bar = 250 μm. **(D)** The mean alveolar linear intercept length in WT and triple n/i/eNOSs^−/−^ mice at 36 weeks after birth. ***P < 0.001 vs. WT mice.

We studied the time course of the progression of spontaneous pulmonary emphysema in the triple n/i/eNOSs^−/−^ mice from 4 to 36 weeks after birth. The micro CT analysis indicated that as early as 4 weeks after birth, a significant reduction in the lung field CT value in the triple n/i/eNOSs^−/−^ mice was noted, and the significant reduction was observed up to 36 weeks after birth throughout the experimental period (Fig. 3A). In the triple n/i/eNOSs^−/−^ mice at 36 weeks after birth, significant increases in the end-expiratory lung volume, the alveolar air-space size, and the mean alveolar linear intercept length were also observed (Fig. 3B–D).

### No change of BALF profile in triple n/i/eNOSs^−/−^ mice

We then investigated the underlying mechanisms for spontaneous pulmonary emphysema in the triple n/i/eNOSs^−/−^ mice. There were no significant differences in the counts of total cells, macrophages, lymphocytes, eosinophils, or basophils in the BALF among 8 genotypes studied (Table 1). Although the neutrophil count in the BALF significantly increased in only the double n/iNOSs^−/−^ mice, the significance of this result is unknown. On the other hand, it is interesting to note that the macrophage and total cell counts in the BALF were the largest in the triple n/i/eNOSs^−/−^ mice despite the lack of statistically significant differences.

**Table 1:** BALF profile in WT, and single, double, and triple NOSs^−/−^ mice.

Mice	Total cell count (× 10^3^/mL)	Macrophage count (× 10^3^/mL)	Lymphocyte count (× 10^3^/mL)	Neutrophil count (× 10^3^/mL)	Eosinophil count (× 10^3^/mL)
WT	52.78 ± 6.93	49.66 ± 6.19	3.02 ± 0.87	0.10 ± 0.10	0.00 ± 0.00
nNOS^−/−^	48.75 ± 5.65	45.07 ± 4.96	2.55 ± 0.57	1.06 ± 0.63	0.00 ± 0.00
iNOS^−/−^	50.63 ± 7.59	46.39 ± 7.01	4.16 ± 0.87	0.08 ± 0.05	0.00 ± 0.00
eNOS^−/−^	56.88 ± 6.47	50.99 ± 5.96	4.91 ± 0.98	1.41 ± 0.44	0.00 ± 0.00
n/iNOSs^−/−^	63.89 ± 5.58	56.59 ± 4.71	3.44 ± 0.70	3.47 ± 1.09***	0.00 ± 0.00
n/eNOSs^−/−^	49.44 ± 5.30	45.82 ± 5.03	3.06 ± 0.59	1.64 ± 0.89	0.04 ± 0.04
i/eNOSs^−/−^	52.50 ± 5.00	49.78 ± 4.71	2.72 ± 0.71	0.00 ± 0.00	0.00 ± 0.00
n/i/eNOSs^−/−^	66.67 ± 4.25	60.90 ± 4.05	3.82 ± 0.37	1.94 ± 0.35	0.00 ± 0.00

n = 8–9, ***P < 0.001 vs. WT mice.

### Mechanisms for spontaneous pulmonary emphysema in triple n/i/eNOSs^−/−^ mice

We performed CAGE sequencing in the lung tissues of the triple n/i/eNOSs^−/−^ mice. We used 4-week-old WT and triple n/i/eNOSs^−/−^ mice based on the results of the time course experiments. We cyclopaedically and quantitatively analyzed 15,728 mouse genes enrolled in the reference mouse genome mm9 database. Among those genes, there were differential expressions of 4717 mRNAs between the lungs of the WT and triple n/i/eNOSs^−/−^ mice (n = 4 each), with statistically significant differences (*P* < 0.05) and greater than 1.2-fold change; 2108 and 2609 mRNAs were significantly up- and down-regulated, respectively, in the lungs of the triple n/i/eNOSs^−/−^ mice as compared with the WT mice. A volcano plot is presented in the Supplementary Fig. S1.

**Figure 4:**
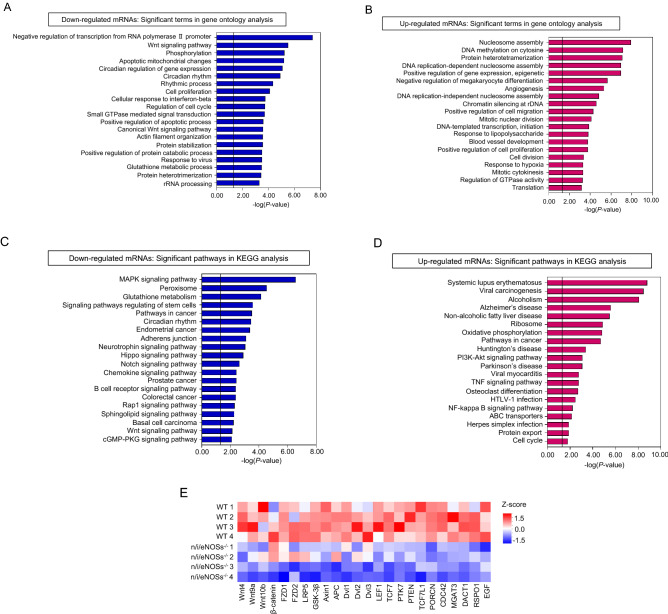
Gene ontology term enrichment analysis and KEGG pathway analysis of differentially expressed genes between the lungs of WT and triple n/i/eNOSs^−/−^ mice. CAGE sequencing identified 4717 mRNAs which were differentially expressed between the lungs of the WT and n/i/eNOSs^−/−^ mice with statistically significant differences (*P* < 0.05) and more than 1.2-fold change (n = 4 each); 2108 and 2609 mRNAs were significantly up- and down-regulated, respectively, in the lungs of the triple n/i/eNOSs^−/−^ mice. Database for Annotation, Visualization, and Integrated Discovery (DAVID) was used. Statistically significant biological process terms in the down-regulated **(A)** and up-regulated mRNAs **(B)** detected by the gene ontology term enrichment analysis. Statistically significant pathways in the down-regulated **(C)** and up-regulated mRNAs **(D)** detected by the KEGG pathway analysis. **(E)** Heat maps of significantly down-regulated genes in the lungs of triple n/i/eNOSs^−/−^ mice as compared with the WT mice. The genes were categorized as Wnt/β-catenin signaling pathway in the gene ontology term enrichment analysis with DAVID. Statistical analysis was performed by Danaform Inc. (Kanagawa, Japan) as follows. The data of mRNA expression levels (gene counts) were corrected by size factors in order to normalize them by the normalization method of trimmed mean of M values (TMM). The corrected data were analyzed by the likelihood ratio test on a negative binomial distribution using edgeR as previously reported^22^.

Gene ontology term enrichment analysis indicated that, in the down-regulated mRNAs in the lungs of the triple n/i/eNOSs^−/−^ mice, statistically significant biological process terms included “Wnt signaling pathway,” “phosphorylation,” “cell proliferation,” “canonical Wnt signaling pathway,” etc. (Fig. 4A, Supplementary Table S1). In the up-regulated mRNAs in the lungs of the triple n/i/eNOSs^−/−^ mice, statistically significant biological process terms included “nucleosome assembly,” “DNA methylation on cytosine,” “protein heterotetramerization,” etc. (Fig. 4B, Supplementary Table S2). Significance of those changes is unknown.

KEGG pathway analysis showed that, in the down-regulated mRNAs, statistically significant pathways included “Notch signaling pathway,” “Wnt signaling pathway,” “cGMP-PKG (protein kinase G) signaling pathway,” etc. (Fig. 4C, Supplementary Table S3). In the up-regulated mRNAs, statistically significant pathways included “PI3K-Akt signaling pathway,” “TNF signaling pathway”, “NF-kappa B signaling pathway,” etc. (Fig. 4D, Supplementary Table S4).

**Figure 5:**
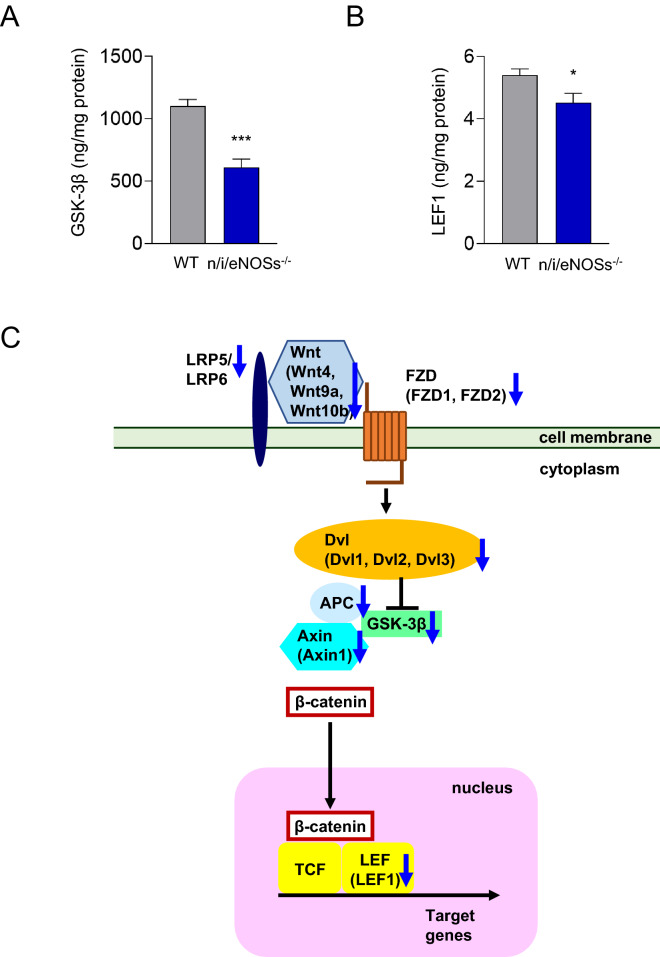
Lung GSK-3β and LEF1 protein expression levels and a schematic diagram showing the down-regulation of the Wnt ligands and Wnt/β-catenin signaling components in the lung of triple n/i/eNOSs^−/−^ mice. **(A)** GSK-3β protein expression levels in the lung (n = 9–10), ***P < 0.001. **(B)** LEF1 protein expression levels in the lung (n = 9–10), *P < 0.05. **(C)** A schematic diagram showing the down-regulation of the Wnt ligands and Wnt/β-catenin signaling components in the lung of triple n/i/eNOSs^−/−^ mice. Blue down arrows indicate the down-regulation. *LRP* lipoprotein receptor-related proteins, *FZD* frizzled, *Dvl* disheveled, *APC* adenomatous polyposis, *GSK-3β* glycogen synthase kinase-3β, *TCF* T cell factor, *LEF* lymphoid enhancer-binding factor.

Since both the gene ontology term enrichment analysis and the KEGG pathway analysis revealed down-regulation of the Wnt signaling pathway in the development of spontaneous pulmonary emphysema in the triple n/i/eNOSs^−/−^ mice, we selected individual genes with statistically significant differences. Three Wnt ligand genes (Wnt4, Wnt9a, and Wnt10b) were significantly reduced, and β-catenin also tended to decrease in the lungs of the triple n/i/eNOS^−/−^ mice (Fig. 4E, Supplementary Fig. S2). Furthermore, expressions of the following ten Wnt/β-catenin signaling component genes were significantly diminished in the lungs of the triple n/i/eNOS^−/−^ mice: frizzled 1 (FZD1), FZD2, lipoprotein receptor-related protein 5 (LRP5), and LRP6, which are cell membrane receptors for the Wnt ligands; GSK-3β, Axin1, adenomatous polyposis (APC), dishevelled 1 (Dvl1), Dvl2, and Dvl3, which are cytoplasmic β-catenin regulators; and LEF1, which is a nucleic transcription factor (Fig. 4E, Supplementary Fig. S3). Protein expression levels of GSK-3β and LEF1 also significantly decreased in the lung of the triple n/i/eNOSs^−/−^ mice as compared with the WT mice (Fig. 5A,B). A schematic diagram indicating the down-regulation of the Wnt ligands and Wnt/β-catenin signaling components in the lung of the triple n/i/eNOSs^−/−^ mice is shown in Fig. 5C.

Other genes that may be related to the mechanisms for spontaneous pulmonary emphysema in the triple n/i/eNOSs^−/−^ mice are presented in Supplementary Figs. S4 and S5. Levels of foxf1 and TCF21, which are reported to be responsible for the development of spontaneous pulmonary emphysema in gene knockout mice^24^, were significantly reduced in the lungs of the triple n/i/eNOSs^−/−^ mice (Supplementary Fig. S4). Levels of histone deacetylase 1 (HDAC1) and sirtuin 1 (SIRT1), which are reported to participate in the mechanisms for experimental pulmonary emphysema, were significantly diminished in the lungs of the triple n/i/eNOSs^−/−^ mice (Supplementary Fig. S5).

## Discussion

### n/i/eNOSs deficiency causes spontaneous pulmonary emphysema in mice in vivo

Under basal conditions (without tobacco smoke exposure or elastase treatment), the triple n/i/eNOSs^−/−^ mice displayed longer mean alveolar linear intercept length, increased alveolar destructive index, reduced lung elastic fiber content, lower lung field CT value, and larger end-expiratory lung volume. These findings were noted in the triple n/i/eNOSs^−/−^ mice as early as 4 weeks after birth immediately after weaning. It has been shown that the extent of reduction of the lung field CT value is correlated with the severity of histopathologically evaluated pulmonary emphysema, and that the extent of increase in the end-expiratory lung volume is correlated with that of histopathologically evaluated alveolar expansion and destruction in pulmonary emphysema^18^. Thus, the present findings suggest that n/i/eNOSs deficiency causes spontaneous pulmonary emphysema in juvenile mice in vivo.

NO synthesized by NOSs stimulates soluble guanylate cyclase (sGC) and generates the second messenger cGMP, which in turn activates PKG and exerts a variety of biological actions. In emphysematous lungs of the triple n/i/eNOSs^−/−^ mice, the cGMP-PKG signaling pathway was down-regulated. Consistent with our findings, it has been reported that cGC and cGMP levels and PKG activity are lowered in the lungs of mice with tobacco smoke-induced pulmonary emphysema^25^, that eNOS and sGC levels are reduced in the lungs of patients with COPD^25,26^, that long-term treatment with either sGC stimulators, inorganic nitrite, or l-arginine prevents pulmonary emphysema induced by tobacco smoke or elastase in mice or pigs^11,27,28^, and that long-term treatment with the sGC stimulator riociguat decreases airway resistance in patients with COPD and pulmonary hypertension^29^. Considering the defective eNOS and sGC levels and the beneficial effects of riociguat in the COPD patients, the present results may have clinical implications.

Spontaneous pulmonary emphysema was noted only in the triple n/i/eNOSs^−/−^ mice, but not in any single or double NOSs^−/−^ mice. The plasma NOx levels were reduced in the order of single, double, and triple NOSs^−/−^ mice, and were considerably preserved in the single NOS^−/−^ and double NOSs^−/−^ mice. Thus, in the single NOS^−/−^ or double NOSs^−/−^ mice, compensatory mechanisms by other NOSs that are not genetically disrupted may operate. Indeed, we previously reported compensatory up-regulation of other NOS isoforms in the single NOS^−/−^ mice^19,30^.

### Wnt/β-catenin signaling pathway possibly mediates the development of spontaneous pulmonary emphysema caused by n/i/eNOSs deficiency in mice

We next examined the underlying mechanisms for spontaneous pulmonary emphysema in the triple n/i/eNOSs^−/−^ mice. There was no difference in the BALF profile among the 8 genotypes studied. We then made cyclopaedic and quantitative comparisons of mRNA expression levels in the lungs of 4-week-old WT and triple n/i/eNOSs^−/−^ mice by the CAGE sequencing with a next-generation sequencer. The CAGE sequencing is a recently developed gene expression analysis technique that is superior to RNA sequencing in terms of quantitativity. Conventional RNA sequencing has high quantitative performance that is comparable to real-time PCR (“gold standard” of mRNA quantitation methods) with 94% of quantitative concordance rate^31^; however, the RNA sequencing protocol includes a PCR amplification step of sample cDNAs, which incurs a reduction of quantitative performance. The CAGE sequencing does not require the PCR amplification step, thereby showing excellent quantitativity^32^. We thus employed the CAGE sequencing in the present study.

In the gene ontology term enrichment analysis and the KEGG pathway analysis, down-regulation of the Wnt signaling pathway in the lungs of the triple n/i/eNOSs^−/−^ mice was detected. mRNA expression levels of three Wnt ligand genes (Wnt4, Wnt9a, and Wnt10b) and ten Wnt/β-catenin signaling component genes were decreased in the lungs of the triple n/i/eNOSs^−/−^ mice. Specifically, down-regulated Wnt/β-catenin signaling component genes were FZD1, FZD2, and LRP5, which are the cell membrane receptors for the Wnt ligands and transmit Wnt signals into the cytoplasm; GSK-3β, Axin1, and APC, which regulate β-catenin amount in the cytoplasm; Dvl1, Dvl2, and Dvl3, which prevent β-catenin degradation by inhibiting its phosphorylation; and LEF1, which binds to β-catenin in the nucleus and acts as a transcription factor^33^. Protein expression levels of GSK-3β and LEF1 were also reduced in the lungs of the triple n/i/eNOSs^−/−^ mice. Previous studies revealed the causal roles of down-regulation of the Wnt/β-catenin signaling in the pathogenesis of spontaneous pulmonary emphysema as follows: first, mice with tobacco smoke- and elastase-induced pulmonary emphysema show reductions of the Wnt ligands and Wnt/β-catenin signaling components^34^; second, activation of the Wnt/β-catenin signaling pathway by treatment with lithium chloride reverses the pulmonary emphysema, along with amelioration of the reductions of the Wnt ligands and Wnt/β-catenin signaling components^34^; third, human patients with a loss-of-function mutation in the Wnt4 gene exhibit lung dysgenesis (e.g. focal dilatation of distal airways), concomitant with reduced Wnt4 levels and enhanced β-catenin degradation^35^. Therefore, it is conceivable that down-regulation of the Wnt/β-catenin signaling pathway possibly mediates the development of spontaneous pulmonary emphysema in the triple n/i/eNOSs^−/−^ mice. We did not confirm that spontaneous pulmonary emphysema in the triple n/i/eNOSs^−/−^ mice was actually caused by down-regulation of this pathway.

Crosstalk between the NO/NOS system and the Wnt/β-catenin pathway has been reported in two previous studies: treatment with a NO donor improves down-regulated Wnt/β-catenin signaling in renal glomeruli of streptozotocin-induced diabetic rats^36^ and iNOS gene overexpression and knockdown attenuates and activates, respectively, the Wnt/β-catenin signaling in human colon and breast cancer cell lines^37^. Consistent with those lines of evidence, n/i/eNOSs deficiency resulted in down-regulation of the Wnt/β-catenin signaling in the lungs of mice. Thus, the NO/NOS system appears to positively regulate the Wnt/β-catenin signaling in general.

### Other possible mechanisms for the development of spontaneous pulmonary emphysema caused by n/i/eNOSs deficiency in mice

At least 10 strains of gene knockout mice that spontaneously develop pulmonary emphysema have thus far been reported^24^. Among the genes knocked out, foxf1 and TCF21 levels were reduced in the lungs of the triple n/i/eNOSs^−/−^ mice, suggesting that the reduced foxf1 and TCF21 levels were possibly involved in the development of spontaneous pulmonary emphysema in the triple n/i/eNOSs^−/−^ mice. It has been indicated that HDAC expression and activity are reduced in the lungs of patients with COPD, and that chronic treatment with a specific HDAC inhibitor, trichostatin A, causes pulmonary emphysema in rats^38^, while HDAC1 levels were decreased in the lungs of the triple n/i/eNOSs^−/−^ mice. It has been reported that expression levels of sirtuin 1 (SIRT1), a gene related to longevity, are lower in the lungs of patients with COPD^39^, and that SIRT1 ablation and overexpression aggravated and ameliorated, respectively, pulmonary emphysema induced by tobacco smoke in mice^40^, while SIRT1 levels diminished in the lungs of the triple n/i/eNOSs^−/−^ mice. Thus, it is likely that decreased HDAC1 and SIRT1 levels were also involved in the development of spontaneous pulmonary emphysema in the triple n/i/eNOSs^−/−^ mice. Another possible mechanism is that elastic fibers might be more fragile in the triple n/i/eNOSs^−/−^ mice.

### Diversity of the roles of the NOS system in the pathogenesis of respiratory diseases

We have been studying the roles of the entire NOS system in respiratory diseases by the use of murine models of bleomycin-induced pulmonary fibrosis, hypoxia-induced pulmonary hypertension, and ovalbumin-induced bronchial asthma. Bleomycin-induced pulmonary fibrosis is exacerbated in the triple n/i/eNOSs^−/−^ mice, but not in any single NOS^−/−^ mice, as compared with the WT mice, suggesting a protective role of the NOS system in pulmonary fibrosis^41^. Hypoxia-induced pulmonary hypertension is deteriorated in the triple n/i/eNOSs^−/−^ mice, and to a lesser extent, in the eNOS^−/−^ mice, as compared with the WT mice, and hypoxia-induced pulmonary hypertension is also aggravated in the WT mice transplanted with triple n/i/eNOSs^−/−^ bone marrow as compared with those with WT bone marrow, suggesting a protective role of the NOS system, specifically in bone marrow cells, in pulmonary hypertension^19^. However, ovalbumin-induced bronchial asthma is conversely more mitigated in triple n/i/eNOSs^−/−^ than in the WT mice, suggesting an opposing detrimental role of the NOS system in bronchial asthma^42^. These results indicate that, even in the same lung, the roles of the NOS system are different in distinct disease states. In the present study, spontaneous pulmonary emphysema was found in the triple n/i/eNOSs^−/−^ mice, suggesting a preventive role of the NOS system in the occurrence of pulmonary emphysema. It, thus, appears that the NOS system plays diverse roles in the pathogenesis of respiratory diseases.

## Conclusions

We were able to demonstrate that complete disruption of all three NOS genes resulted in the development of spontaneous pulmonary emphysema in juvenile mice in vivo possibly through down-regulation of the Wnt/β-catenin signaling pathway. Our findings should contribute to a better understanding of the molecular basis of pulmonary emphysema for which there is no definitive treatment.

## Supplementary Information

Supplementary Information.
