# Supplementary material for: Spontaneous pulmonary emphysema in mice lacking all three nitric oxide synthase isoforms
Source: Sci Rep. 2021 Nov 11;11:22088. doi: 10.1038/s41598-021-01453-6 (PMC8586362; doi:10.1038/s41598-021-01453-6)
Supplement: Supplementary file 1 — Supplementary Information. [file 41598_2021_1453_MOESM1_ESM.docx]

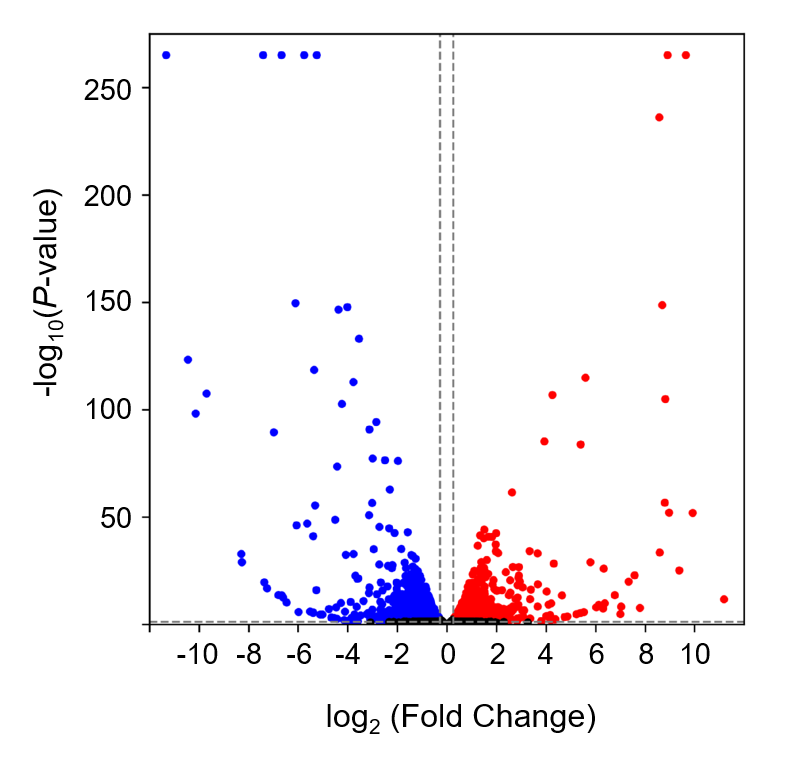


Supplementary Figure E1


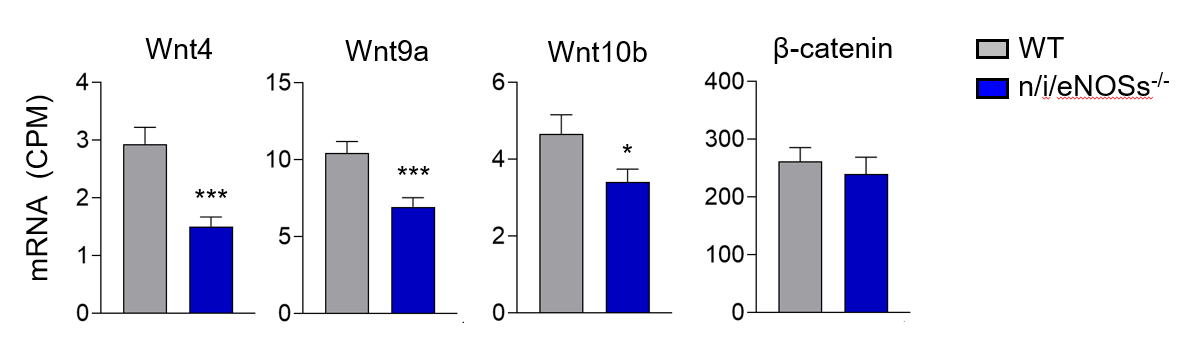


Supplementary Figure E2


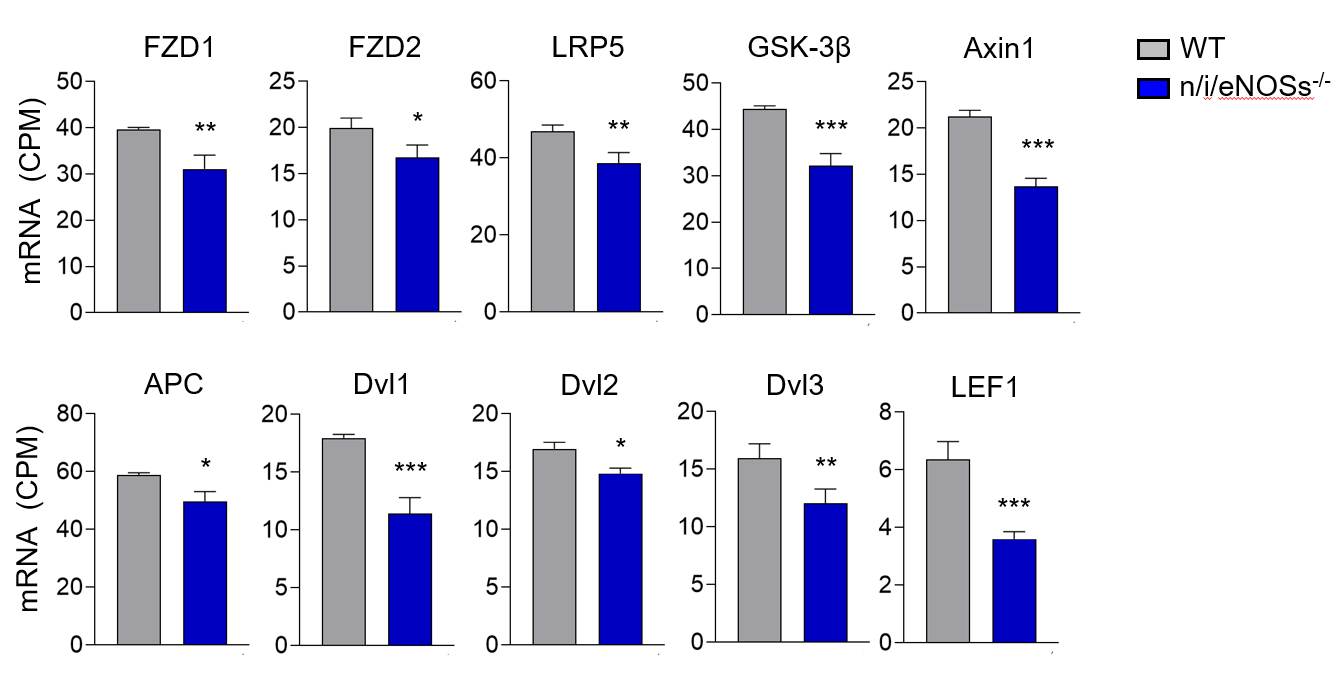


Supplementary Figure E3


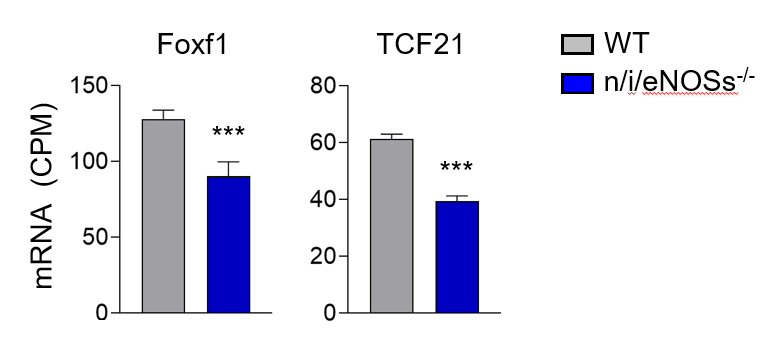


Supplementary Figure E4


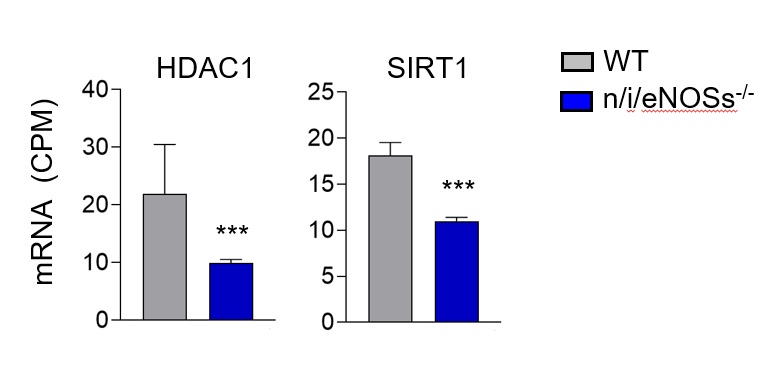


Supplementary Figure E5

# Supplementary Figure Legends

**Supplementary Figure E1**

**A volcano plot for visualizing the distribution of genes with variable expression in CAGE sequencing.** The vertical and horizontal axes indicate statistical significance (*P*-value) and magnitude of change (fold change), respectively.

## Supplementary Figures E2

**Significantly down-regulated genes of Wnt ligands and β-catenin in the lung of triple n/i/eNOSs^-/-^ mice.** **P*<0.05 and ****P*<0.001 vs. WT mice; CPM: counts per million.

**Supplementary Figures E3**

**Significantly down-regulated Wnt/β-catenin signaling component genes in the lung of triple n/i/eNOSs^-/-^ mice.** **P*<0.05, ***P*<0.01, and ****P*<0.001 vs. WT mice.

**Supplementary Figures E4**

**mRNA expression levels of foxf1 and TCF21, which are reported to be responsible for spontaneous pulmonary emphysema in gene knockout mice.** ****P*<0.001 vs. WT mice; CPM: counts per million.

**Supplementary Figures E5**

**mRNA expression levels of histone deacetylase 1 (HDAC1) and sirtuin 1 (SIRT1), which are reported to participate in the mechanisms for experimental pulmonary emphysema.** ****P*<0.001 vs. WT mice.

**Supplementary Table E1**

**Statistically significant biological process terms in the down-regulated mRNAs in the lungs of the triple n/i/eNOSs^-/-^ mice as compared with the WT mice detected by gene ontology term enrichment analysis.**

| GO Term | | *P* Value |
| --- | --- | --- |
| GO:0000122 | negative regulation of transcription from RNA polymerase II promoter | 3.90E-08 |
| GO:0016055 | Wnt signaling pathway | 2.96E-06 |
| GO:0016310 | phosphorylation | 5.64E-06 |
| GO:0008637 | apoptotic mitochondrial changes | 6.18E-06 |
| GO:0032922 | circadian regulation of gene expression | 8.07E-06 |
| GO:0007623 | circadian rhythm | 1.20E-05 |
| GO:0048511 | rhythmic process | 4.30E-05 |
| GO:0008283 | cell proliferation | 7.82E-05 |
| GO:0035458 | cellular response to interferon-beta | 1.76E-04 |
| GO:0051726 | regulation of cell cycle | 1.81E-04 |
| GO:0007264 | small GTPase mediated signal transduction | 1.93E-04 |
| GO:0043065 | positive regulation of apoptotic process | 2.50E-04 |
| GO:0060070 | canonical Wnt signaling pathway | 2.59E-04 |
| GO:0007015 | actin filament organization | 2.67E-04 |
| GO:0050821 | protein stabilization | 2.74E-04 |
| GO:0045732 | positive regulation of protein catabolic process | 3.15E-04 |
| GO:0009615 | response to virus | 3.16E-04 |
| GO:0006749 | glutathione metabolic process | 3.41E-04 |
| GO:0070208 | protein heterotrimerization | 3.61E-04 |
| GO:0006364 | rRNA processing | 5.04E-04 |
| GO:0003341 | cilium movement | 5.97E-04 |
| GO:0006468 | protein phosphorylation | 6.04E-04 |
| GO:0031397 | negative regulation of protein ubiquitination | 7.02E-04 |
| GO:0043153 | entrainment of circadian clock by photoperiod | 8.03E-04 |
| GO:0002376 | immune system process | 8.63E-04 |
| GO:0006351 | transcription, DNA-templated | 8.93E-04 |
| GO:0035556 | intracellular signal transduction | 9.53E-04 |
| GO:0030177 | positive regulation of Wnt signaling pathway | 0.00100663 |
| GO:0045944 | positive regulation of transcription from RNA polymerase II promoter | 0.001043183 |
| GO:0090179 | planar cell polarity pathway involved in neural tube closure | 0.001142701 |
| GO:0071902 | positive regulation of protein serine/threonine kinase activity | 0.001166073 |
| GO:0016337 | single organismal cell-cell adhesion | 0.001565708 |
| GO:0006915 | apoptotic process | 0.001583289 |
| GO:0043124 | negative regulation of I-kappaB kinase/NF-kappaB signaling | 0.001623475 |
| GO:0046632 | alpha-beta T cell differentiation | 0.001967549 |
| GO:0042752 | regulation of circadian rhythm | 0.002130646 |
| GO:0042127 | regulation of cell proliferation | 0.002239717 |
| GO:0050680 | negative regulation of epithelial cell proliferation | 0.002612091 |
| GO:0016567 | protein ubiquitination | 0.002741177 |
| GO:0006487 | protein N-linked glycosylation | 0.002961357 |
| GO:0021591 | ventricular system development | 0.00301274 |
| GO:0055072 | iron ion homeostasis | 0.003096587 |
| GO:0060048 | cardiac muscle contraction | 0.003116128 |
| GO:0055114 | oxidation-reduction process | 0.00313963 |
| GO:0051091 | positive regulation of sequence-specific DNA binding transcription factor activity | 0.003171775 |
| GO:0051597 | response to methylmercury | 0.00317908 |
| GO:0090263 | positive regulation of canonical Wnt signaling pathway | 0.003249011 |
| GO:0032091 | negative regulation of protein binding | 0.003453167 |
| GO:0051289 | protein homotetramerization | 0.003497455 |
| GO:0030030 | cell projection organization | 0.003643661 |
| GO:0030178 | negative regulation of Wnt signaling pathway | 0.004279493 |
| GO:0016571 | histone methylation | 0.004966646 |
| GO:0071353 | cellular response to interleukin-4 | 0.004966646 |
| GO:0007507 | heart development | 0.00499767 |
| GO:0006357 | regulation of transcription from RNA polymerase II promoter | 0.005132234 |
| GO:0006979 | response to oxidative stress | 0.005237393 |
| GO:0060828 | regulation of canonical Wnt signaling pathway | 0.005503139 |
| GO:0007049 | cell cycle | 0.005551542 |
| GO:0039694 | viral RNA genome replication | 0.00583496 |
| GO:0042542 | response to hydrogen peroxide | 0.005873348 |
| GO:0043161 | proteasome-mediated ubiquitin-dependent protein catabolic process | 0.005947677 |
| GO:0071260 | cellular response to mechanical stimulus | 0.006010276 |
| GO:0008285 | negative regulation of cell proliferation | 0.006166762 |
| GO:0050873 | brown fat cell differentiation | 0.006685592 |
| GO:0030855 | epithelial cell differentiation | 0.008314943 |
| GO:0033138 | positive regulation of peptidyl-serine phosphorylation | 0.008577572 |
| GO:0001657 | ureteric bud development | 0.009145055 |
| GO:0030511 | positive regulation of transforming growth factor beta receptor signaling pathway | 0.009279979 |
| GO:0006270 | DNA replication initiation | 0.009279979 |
| GO:0001525 | angiogenesis | 0.009286977 |
| GO:0006942 | regulation of striated muscle contraction | 0.009619962 |
| GO:0006782 | protoporphyrinogen IX biosynthetic process | 0.009642124 |
| GO:0033129 | positive regulation of histone phosphorylation | 0.009642124 |
| GO:0045814 | negative regulation of gene expression, epigenetic | 0.009642124 |
| GO:0042832 | defense response to protozoan | 0.009878443 |
| GO:0050775 | positive regulation of dendrite morphogenesis | 0.009878443 |
| GO:0046328 | regulation of JNK cascade | 0.010445839 |
| GO:0017015 | regulation of transforming growth factor beta receptor signaling pathway | 0.010445839 |
| GO:0007010 | cytoskeleton organization | 0.01061682 |
| GO:0046685 | response to arsenic-containing substance | 0.010952614 |
| GO:0010839 | negative regulation of keratinocyte proliferation | 0.010952614 |
| GO:0034612 | response to tumor necrosis factor | 0.011752462 |
| GO:0030336 | negative regulation of cell migration | 0.012937716 |
| GO:0046928 | regulation of neurotransmitter secretion | 0.013631452 |
| GO:0006164 | purine nucleotide biosynthetic process | 0.013631452 |
| GO:0040014 | regulation of multicellular organism growth | 0.014224381 |
| GO:0006094 | gluconeogenesis | 0.014667377 |
| GO:0010941 | regulation of cell death | 0.014758507 |
| GO:0036158 | outer dynein arm assembly | 0.01504192 |
| GO:0006165 | nucleoside diphosphate phosphorylation | 0.01504192 |
| GO:0001934 | positive regulation of protein phosphorylation | 0.015576432 |
| GO:0043547 | positive regulation of GTPase activity | 0.016280119 |
| GO:0008152 | metabolic process | 0.016533237 |
| GO:0060548 | negative regulation of cell death | 0.01729879 |
| GO:0090630 | activation of GTPase activity | 0.017459541 |
| GO:0019933 | cAMP-mediated signaling | 0.017706912 |
| GO:0019216 | regulation of lipid metabolic process | 0.017706912 |
| GO:0033211 | adiponectin-activated signaling pathway | 0.017754772 |
| GO:0006189 | 'de novo' IMP biosynthetic process | 0.017754772 |
| GO:0035020 | regulation of Rac protein signal transduction | 0.017754772 |
| GO:0010669 | epithelial structure maintenance | 0.017754772 |
| GO:2001243 | negative regulation of intrinsic apoptotic signaling pathway | 0.018062633 |
| GO:0043154 | negative regulation of cysteine-type endopeptidase activity involved in apoptotic process | 0.019641526 |
| GO:0051898 | negative regulation of protein kinase B signaling | 0.019760688 |
| GO:0030163 | protein catabolic process | 0.021052857 |
| GO:1903827 | regulation of cellular protein localization | 0.021306867 |
| GO:0071407 | cellular response to organic cyclic compound | 0.021784918 |
| GO:0035690 | cellular response to drug | 0.021784918 |
| GO:0010719 | negative regulation of epithelial to mesenchymal transition | 0.021912293 |
| GO:0031668 | cellular response to extracellular stimulus | 0.021912293 |
| GO:0045599 | negative regulation of fat cell differentiation | 0.024186489 |
| GO:0016477 | cell migration | 0.024764242 |
| GO:0044458 | motile cilium assembly | 0.026434061 |
| GO:0042113 | B cell activation | 0.026434061 |
| GO:0030334 | regulation of cell migration | 0.026866991 |
| GO:0048041 | focal adhesion assembly | 0.027101202 |
| GO:0031647 | regulation of protein stability | 0.027482364 |
| GO:0007409 | axonogenesis | 0.027743874 |
| GO:1901215 | negative regulation of neuron death | 0.027977441 |
| GO:0006629 | lipid metabolic process | 0.028028453 |
| GO:0010976 | positive regulation of neuron projection development | 0.028503417 |
| GO:0006620 | posttranslational protein targeting to membrane | 0.028686541 |
| GO:0006595 | polyamine metabolic process | 0.028686541 |
| GO:0006930 | substrate-dependent cell migration, cell extension | 0.028686541 |
| GO:0050869 | negative regulation of B cell activation | 0.028686541 |
| GO:0007215 | glutamate receptor signaling pathway | 0.028686541 |
| GO:0043496 | regulation of protein homodimerization activity | 0.028686541 |
| GO:0048015 | phosphatidylinositol-mediated signaling | 0.029209704 |
| GO:0006511 | ubiquitin-dependent protein catabolic process | 0.029276115 |
| GO:0036159 | inner dynein arm assembly | 0.02937364 |
| GO:0010614 | negative regulation of cardiac muscle hypertrophy | 0.02937364 |
| GO:0016579 | protein deubiquitination | 0.029713115 |
| GO:0009113 | purine nucleobase biosynthetic process | 0.030324429 |
| GO:0007399 | nervous system development | 0.031400077 |
| GO:0048535 | lymph node development | 0.031472727 |
| GO:0010975 | regulation of neuron projection development | 0.031472727 |
| GO:0045727 | positive regulation of translation | 0.031515809 |
| GO:0017144 | drug metabolic process | 0.032998592 |
| GO:0046329 | negative regulation of JNK cascade | 0.03304161 |
| GO:0030574 | collagen catabolic process | 0.03304161 |
| GO:0030041 | actin filament polymerization | 0.03304161 |
| GO:0051924 | regulation of calcium ion transport | 0.033976153 |
| GO:0007219 | Notch signaling pathway | 0.034331284 |
| GO:0032436 | positive regulation of proteasomal ubiquitin-dependent protein catabolic process | 0.034739521 |
| GO:0006635 | fatty acid beta-oxidation | 0.035210096 |
| GO:0007568 | aging | 0.035913349 |
| GO:0045214 | sarcomere organization | 0.037115827 |
| GO:0005975 | carbohydrate metabolic process | 0.037434036 |
| GO:0030036 | actin cytoskeleton organization | 0.03781362 |
| GO:0070373 | negative regulation of ERK1 and ERK2 cascade | 0.038583008 |
| GO:0032880 | regulation of protein localization | 0.038583008 |
| GO:0022407 | regulation of cell-cell adhesion | 0.039009721 |
| GO:0060412 | ventricular septum morphogenesis | 0.039243292 |
| GO:0001503 | ossification | 0.039336255 |
| GO:0010906 | regulation of glucose metabolic process | 0.039764499 |
| GO:0010800 | positive regulation of peptidyl-threonine phosphorylation | 0.039764499 |
| GO:0031532 | actin cytoskeleton reorganization | 0.040089898 |
| GO:0045087 | innate immune response | 0.040963486 |
| GO:0006783 | heme biosynthetic process | 0.041053147 |
| GO:2000045 | regulation of G1/S transition of mitotic cell cycle | 0.041053147 |
| GO:0071901 | negative regulation of protein serine/threonine kinase activity | 0.04239749 |
| GO:0060603 | mammary gland duct morphogenesis | 0.04239749 |
| GO:0009635 | response to herbicide | 0.04239749 |
| GO:2000480 | negative regulation of cAMP-dependent protein kinase activity | 0.04239749 |
| GO:0042754 | negative regulation of circadian rhythm | 0.04239749 |
| GO:0038180 | nerve growth factor signaling pathway | 0.04239749 |
| GO:0007183 | SMAD protein complex assembly | 0.04239749 |
| GO:0030858 | positive regulation of epithelial cell differentiation | 0.04239749 |
| GO:0050688 | regulation of defense response to virus | 0.04239749 |
| GO:0033081 | regulation of T cell differentiation in thymus | 0.04239749 |
| GO:0050687 | negative regulation of defense response to virus | 0.04239749 |
| GO:1900744 | regulation of p38MAPK cascade | 0.04239749 |
| GO:0070555 | response to interleukin-1 | 0.043385266 |
| GO:2000134 | negative regulation of G1/S transition of mitotic cell cycle | 0.043385266 |
| GO:0006974 | cellular response to DNA damage stimulus | 0.044851949 |
| GO:0045893 | positive regulation of transcription, DNA-templated | 0.04492973 |
| GO:0005977 | glycogen metabolic process | 0.045028158 |
| GO:0035019 | somatic stem cell population maintenance | 0.045409478 |
| GO:0007266 | Rho protein signal transduction | 0.045409478 |
| GO:0034644 | cellular response to UV | 0.045409478 |
| GO:0030317 | sperm motility | 0.047146089 |
| GO:0021987 | cerebral cortex development | 0.047146089 |
| GO:0006006 | glucose metabolic process | 0.047146089 |
| GO:0060716 | labyrinthine layer blood vessel development | 0.047293636 |
| GO:0006355 | regulation of transcription, DNA-templated | 0.047372179 |
| GO:0042147 | retrograde transport, endosome to Golgi | 0.048844677 |
| GO:0006470 | protein dephosphorylation | 0.049151533 |
| GO:0045892 | negative regulation of transcription, DNA-templated | 0.049561486 |

**Supplementary Table E2**

**Statistically significant biological process terms in the up-regulated mRNAs in the lungs of the triple n/i/eNOSs^-/-^ mice as compared with the WT mice detected by gene ontology term enrichment analysis.**

| GO Term | | *P* Value |
| --- | --- | --- |
| GO:0006334 | nucleosome assembly | 1.18E-08 |
| GO:0032776 | DNA methylation on cytosine | 6.73E-08 |
| GO:0051290 | protein heterotetramerization | 7.98E-08 |
| GO:0006335 | DNA replication-dependent nucleosome assembly | 1.08E-07 |
| GO:0045815 | positive regulation of gene expression, epigenetic | 1.08E-07 |
| GO:0045653 | negative regulation of megakaryocyte differentiation | 2.17E-06 |
| GO:0001525 | angiogenesis | 4.75E-06 |
| GO:0006336 | DNA replication-independent nucleosome assembly | 1.39E-05 |
| GO:0000183 | chromatin silencing at rDNA | 2.44E-05 |
| GO:0030335 | positive regulation of cell migration | 4.94E-05 |
| GO:0007067 | mitotic nuclear division | 7.67E-05 |
| GO:0006352 | DNA-templated transcription, initiation | 1.26E-04 |
| GO:0032496 | response to lipopolysaccharide | 1.47E-04 |
| GO:0001568 | blood vessel development | 1.63E-04 |
| GO:0008284 | positive regulation of cell proliferation | 1.65E-04 |
| GO:0051301 | cell division | 4.12E-04 |
| GO:0001666 | response to hypoxia | 4.60E-04 |
| GO:0000281 | mitotic cytokinesis | 4.80E-04 |
| GO:0043087 | regulation of GTPase activity | 5.40E-04 |
| GO:0006412 | translation | 6.55E-04 |
| GO:0008285 | negative regulation of cell proliferation | 7.51E-04 |
| GO:0002227 | innate immune response in mucosa | 0.001023629 |
| GO:0098792 | xenophagy | 0.001691759 |
| GO:0010243 | response to organonitrogen compound | 0.001866114 |
| GO:0007049 | cell cycle | 0.001991031 |
| GO:0045740 | positive regulation of DNA replication | 0.00208475 |
| GO:0006954 | inflammatory response | 0.002155528 |
| GO:0007507 | heart development | 0.002677216 |
| GO:0070374 | positive regulation of ERK1 and ERK2 cascade | 0.003024765 |
| GO:0000132 | establishment of mitotic spindle orientation | 0.003165314 |
| GO:0045893 | positive regulation of transcription, DNA-templated | 0.003517337 |
| GO:0006810 | transport | 0.003821977 |
| GO:0003151 | outflow tract morphogenesis | 0.003881805 |
| GO:0050768 | negative regulation of neurogenesis | 0.004029347 |
| GO:0071236 | cellular response to antibiotic | 0.00405728 |
| GO:0006488 | dolichol-linked oligosaccharide biosynthetic process | 0.00405728 |
| GO:0071260 | cellular response to mechanical stimulus | 0.004124245 |
| GO:0060326 | cell chemotaxis | 0.004124245 |
| GO:0019731 | antibacterial humoral response | 0.004355014 |
| GO:0009966 | regulation of signal transduction | 0.004359683 |
| GO:0048286 | lung alveolus development | 0.004891645 |
| GO:0002230 | positive regulation of defense response to virus by host | 0.0049007 |
| GO:0008016 | regulation of heart contraction | 0.005060769 |
| GO:0035987 | endodermal cell differentiation | 0.005060769 |
| GO:0006970 | response to osmotic stress | 0.005576772 |
| GO:0007059 | chromosome segregation | 0.005620685 |
| GO:0002376 | immune system process | 0.00579823 |
| GO:0048146 | positive regulation of fibroblast proliferation | 0.006049652 |
| GO:0006915 | apoptotic process | 0.00626478 |
| GO:0043065 | positive regulation of apoptotic process | 0.006437216 |
| GO:0009611 | response to wounding | 0.006835049 |
| GO:0045736 | negative regulation of cyclin-dependent protein serine/threonine kinase activity | 0.00714457 |
| GO:0016311 | dephosphorylation | 0.00741607 |
| GO:0007512 | adult heart development | 0.007476158 |
| GO:0007568 | aging | 0.007668365 |
| GO:0001701 | in utero embryonic development | 0.007783571 |
| GO:0050830 | defense response to Gram-positive bacterium | 0.008466736 |
| GO:0001570 | vasculogenesis | 0.00872039 |
| GO:0007155 | cell adhesion | 0.008813989 |
| GO:0000070 | mitotic sister chromatid segregation | 0.009007554 |
| GO:0007346 | regulation of mitotic cell cycle | 0.00904266 |
| GO:0032964 | collagen biosynthetic process | 0.009114278 |
| GO:0051272 | positive regulation of cellular component movement | 0.009302026 |
| GO:2000379 | positive regulation of reactive oxygen species metabolic process | 0.011240369 |
| GO:0043552 | positive regulation of phosphatidylinositol 3-kinase activity | 0.011240369 |
| GO:0030199 | collagen fibril organization | 0.012459656 |
| GO:0045722 | positive regulation of gluconeogenesis | 0.013059022 |
| GO:0045086 | positive regulation of interleukin-2 biosynthetic process | 0.013059022 |
| GO:0033137 | negative regulation of peptidyl-serine phosphorylation | 0.013728508 |
| GO:0060325 | face morphogenesis | 0.01448465 |
| GO:0032466 | negative regulation of cytokinesis | 0.014983567 |
| GO:0002467 | germinal center formation | 0.014983567 |
| GO:0048842 | positive regulation of axon extension involved in axon guidance | 0.014983567 |
| GO:0032147 | activation of protein kinase activity | 0.015827131 |
| GO:0018108 | peptidyl-tyrosine phosphorylation | 0.015917088 |
| GO:0042176 | regulation of protein catabolic process | 0.01619916 |
| GO:1901224 | positive regulation of NIK/NF-kappaB signaling | 0.01619916 |
| GO:0042346 | positive regulation of NF-kappaB import into nucleus | 0.01619916 |
| GO:0055117 | regulation of cardiac muscle contraction | 0.01619916 |
| GO:0000079 | regulation of cyclin-dependent protein serine/threonine kinase activity | 0.01619916 |
| GO:0070301 | cellular response to hydrogen peroxide | 0.016548163 |
| GO:0043066 | negative regulation of apoptotic process | 0.016958611 |
| GO:0050731 | positive regulation of peptidyl-tyrosine phosphorylation | 0.017508247 |
| GO:0060968 | regulation of gene silencing | 0.017658344 |
| GO:0007076 | mitotic chromosome condensation | 0.017658344 |
| GO:0035264 | multicellular organism growth | 0.018514485 |
| GO:0010718 | positive regulation of epithelial to mesenchymal transition | 0.018558157 |
| GO:0060856 | establishment of blood-brain barrier | 0.019065715 |
| GO:0043331 | response to dsRNA | 0.019065715 |
| GO:0038091 | positive regulation of cell proliferation by VEGF-activated platelet derived growth factor receptor signaling pathway | 0.019065715 |
| GO:0035284 | brain segmentation | 0.019065715 |
| GO:0006936 | muscle contraction | 0.019137845 |
| GO:0042127 | regulation of cell proliferation | 0.019539518 |
| GO:0015031 | protein transport | 0.019790813 |
| GO:0006885 | regulation of pH | 0.019946109 |
| GO:0001578 | microtubule bundle formation | 0.019946109 |
| GO:0090398 | cellular senescence | 0.020173589 |
| GO:0006986 | response to unfolded protein | 0.02160139 |
| GO:0043407 | negative regulation of MAP kinase activity | 0.021984175 |
| GO:0002315 | marginal zone B cell differentiation | 0.022527756 |
| GO:0014009 | glial cell proliferation | 0.022527756 |
| GO:0008283 | cell proliferation | 0.022836025 |
| GO:0030949 | positive regulation of vascular endothelial growth factor receptor signaling pathway | 0.023148055 |
| GO:0051896 | regulation of protein kinase B signaling | 0.023148055 |
| GO:0009791 | post-embryonic development | 0.023675286 |
| GO:0018105 | peptidyl-serine phosphorylation | 0.024001467 |
| GO:0006979 | response to oxidative stress | 0.024001467 |
| GO:0006357 | regulation of transcription from RNA polymerase II promoter | 0.024752677 |
| GO:0010634 | positive regulation of epithelial cell migration | 0.02497767 |
| GO:0048008 | platelet-derived growth factor receptor signaling pathway | 0.02497767 |
| GO:0002474 | antigen processing and presentation of peptide antigen via MHC class I | 0.02497767 |
| GO:0045944 | positive regulation of transcription from RNA polymerase II promoter | 0.02514117 |
| GO:0051897 | positive regulation of protein kinase B signaling | 0.026563793 |
| GO:0045444 | fat cell differentiation | 0.026865533 |
| GO:0007173 | epidermal growth factor receptor signaling pathway | 0.028293696 |
| GO:0000910 | cytokinesis | 0.028695838 |
| GO:0048661 | positive regulation of smooth muscle cell proliferation | 0.029334525 |
| GO:0000188 | inactivation of MAPK activity | 0.02956303 |
| GO:0006977 | DNA damage response, signal transduction by p53 class mediator resulting in cell cycle arrest | 0.031762994 |
| GO:0051926 | negative regulation of calcium ion transport | 0.031762994 |
| GO:0060982 | coronary artery morphogenesis | 0.031762994 |
| GO:0031340 | positive regulation of vesicle fusion | 0.031762994 |
| GO:0034097 | response to cytokine | 0.031963212 |
| GO:0051092 | positive regulation of NF-kappaB transcription factor activity | 0.032557427 |
| GO:0021762 | substantia nigra development | 0.03277066 |
| GO:0010977 | negative regulation of neuron projection development | 0.034673409 |
| GO:0031175 | neuron projection development | 0.035412523 |
| GO:0008360 | regulation of cell shape | 0.035412523 |
| GO:0007178 | transmembrane receptor protein serine/threonine kinase signaling pathway | 0.035714673 |
| GO:0038084 | vascular endothelial growth factor signaling pathway | 0.036064057 |
| GO:0035633 | maintenance of blood-brain barrier | 0.036064057 |
| GO:1990966 | ATP generation from poly-ADP-D-ribose | 0.036064057 |
| GO:0045404 | positive regulation of interleukin-4 biosynthetic process | 0.036064057 |
| GO:0000920 | cell separation after cytokinesis | 0.036925321 |
| GO:0030261 | chromosome condensation | 0.036925321 |
| GO:0010666 | positive regulation of cardiac muscle cell apoptotic process | 0.036925321 |
| GO:0031100 | organ regeneration | 0.03738642 |
| GO:0045766 | positive regulation of angiogenesis | 0.038570225 |
| GO:0045599 | negative regulation of fat cell differentiation | 0.039937894 |
| GO:0016477 | cell migration | 0.040498669 |
| GO:0000209 | protein polyubiquitination | 0.04160372 |
| GO:0009409 | response to cold | 0.042035004 |
| GO:0045216 | cell-cell junction organization | 0.042167655 |
| GO:0033628 | regulation of cell adhesion mediated by integrin | 0.042664519 |
| GO:1902188 | positive regulation of viral release from host cell | 0.042664519 |
| GO:0034501 | protein localization to kinetochore | 0.042664519 |
| GO:0001755 | neural crest cell migration | 0.044436166 |
| GO:0046777 | protein autophosphorylation | 0.045030743 |
| GO:2000147 | positive regulation of cell motility | 0.045244787 |
| GO:0006469 | negative regulation of protein kinase activity | 0.046209399 |
| GO:0000086 | G2/M transition of mitotic cell cycle | 0.04920038 |
| GO:0032024 | positive regulation of insulin secretion | 0.049254894 |
| GO:0048844 | artery morphogenesis | 0.049277441 |
| GO:0032456 | endocytic recycling | 0.049277441 |

**Supplementary Table E3**

**Statistically significant pathways in the down-regulated mRNAs in the lungs of the triple n/i/eNOSs^-/-^ mice as compared with the WT mice detected by the KEGG pathway analysis.**

| Term | | *P* Value |
| --- | --- | --- |
| mmu04010 | MAPK signaling pathway | 2.59E-07 |
| mmu04146 | Peroxisome | 2.74E-05 |
| mmu00480 | Glutathione metabolism | 6.79E-05 |
| mmu04550 | Signaling pathways regulating pluripotency of stem cells | 2.59E-04 |
| mmu05200 | Pathways in cancer | 3.10E-04 |
| mmu04710 | Circadian rhythm | 3.45E-04 |
| mmu05213 | Endometrial cancer | 4.19E-04 |
| mmu04520 | Adherens junction | 7.89E-04 |
| mmu04722 | Neurotrophin signaling pathway | 8.93E-04 |
| mmu04390 | Hippo signaling pathway | 0.001256712 |
| mmu04330 | Notch signaling pathway | 0.002320974 |
| mmu04062 | Chemokine signaling pathway | 0.003541304 |
| mmu05215 | Prostate cancer | 0.003610686 |
| mmu04662 | B cell receptor signaling pathway | 0.004042778 |
| mmu05210 | Colorectal cancer | 0.004119419 |
| mmu04015 | Rap1 signaling pathway | 0.004821226 |
| mmu04071 | Sphingolipid signaling pathway | 0.00523429 |
| mmu05217 | Basal cell carcinoma | 0.005768426 |
| mmu04310 | Wnt signaling pathway | 0.007263788 |
| mmu04022 | cGMP-PKG signaling pathway | 0.007926811 |
| mmu04660 | T cell receptor signaling pathway | 0.007960509 |
| mmu05160 | Hepatitis C | 0.008714697 |
| mmu01100 | Metabolic pathways | 0.009171467 |
| mmu00980 | Metabolism of xenobiotics by cytochrome P450 | 0.010337709 |
| mmu04640 | Hematopoietic cell lineage | 0.012500929 |
| mmu04910 | Insulin signaling pathway | 0.012504072 |
| mmu00982 | Drug metabolism - cytochrome P450 | 0.013538343 |
| mmu04270 | Vascular smooth muscle contraction | 0.013756435 |
| mmu04540 | Gap junction | 0.014003302 |
| mmu01130 | Biosynthesis of antibiotics | 0.014078928 |
| mmu05211 | Renal cell carcinoma | 0.017455115 |
| mmu03008 | Ribosome biogenesis in eukaryotes | 0.021084246 |
| mmu04510 | Focal adhesion | 0.023713679 |
| mmu04978 | Mineral absorption | 0.02617979 |
| mmu04916 | Melanogenesis | 0.026241127 |
| mmu04120 | Ubiquitin mediated proteolysis | 0.026455236 |
| mmu04261 | Adrenergic signaling in cardiomyocytes | 0.026455236 |
| mmu04152 | AMPK signaling pathway | 0.027492861 |
| mmu05220 | Chronic myeloid leukemia | 0.02780196 |
| mmu05202 | Transcriptional misregulation in cancer | 0.028411746 |
| mmu04917 | Prolactin signaling pathway | 0.030976995 |
| mmu05340 | Primary immunodeficiency | 0.031526794 |
| mmu00051 | Fructose and mannose metabolism | 0.031526794 |
| mmu04370 | VEGF signaling pathway | 0.032753876 |
| mmu04810 | Regulation of actin cytoskeleton | 0.034237351 |
| mmu04066 | HIF-1 signaling pathway | 0.034366003 |
| mmu04611 | Platelet activation | 0.034735643 |
| mmu04912 | GnRH signaling pathway | 0.03479107 |
| mmu04110 | Cell cycle | 0.035513732 |
| mmu04730 | Long-term depression | 0.036768347 |
| mmu04664 | Fc epsilon RI signaling pathway | 0.037636327 |
| mmu00510 | N-Glycan biosynthesis | 0.04320858 |
| mmu05223 | Non-small cell lung cancer | 0.044847659 |
| mmu05221 | Acute myeloid leukemia | 0.044847659 |
| mmu05205 | Proteoglycans in cancer | 0.046432554 |
| mmu04962 | Vasopressin-regulated water reabsorption | 0.046528294 |
| mmu00250 | Alanine, aspartate and glutamate metabolism | 0.049728153 |

**Supplementary Table E4**

**Statistically significant terms in the up-regulated mRNAs in the lungs of the triple n/i/eNOSs^-/-^ mice as compared with the WT mice detected by the KEGG pathway analysis.**

| Term | | *P* Value |
| --- | --- | --- |
| mmu05322 | Systemic lupus erythematosus | 1.53E-09 |
| mmu05203 | Viral carcinogenesis | 3.27E-09 |
| mmu05034 | Alcoholism | 8.03E-09 |
| mmu05010 | Alzheimer's disease | 2.62E-06 |
| mmu04932 | Non-alcoholic fatty liver disease (NAFLD) | 3.23E-06 |
| mmu03010 | Ribosome | 1.25E-05 |
| mmu00190 | Oxidative phosphorylation | 1.48E-05 |
| mmu05200 | Pathways in cancer | 1.93E-05 |
| mmu05016 | Huntington's disease | 4.11E-04 |
| mmu04151 | PI3K-Akt signaling pathway | 7.82E-04 |
| mmu05012 | Parkinson's disease | 8.16E-04 |
| mmu05416 | Viral myocarditis | 0.001669372 |
| mmu04668 | TNF signaling pathway | 0.001694731 |
| mmu04380 | Osteoclast differentiation | 0.001985022 |
| mmu05166 | HTLV-I infection | 0.003336278 |
| mmu04064 | NF-kappa B signaling pathway | 0.00593473 |
| mmu02010 | ABC transporters | 0.007136006 |
| mmu05168 | Herpes simplex infection | 0.012582016 |
| mmu03060 | Protein export | 0.01304288 |
| mmu04110 | Cell cycle | 0.015813573 |
| mmu05222 | Small cell lung cancer | 0.018159642 |
| mmu05211 | Renal cell carcinoma | 0.018160274 |
| mmu05161 | Hepatitis B | 0.022491465 |
| mmu05142 | Chagas disease (American trypanosomiasis) | 0.022627803 |
| mmu05218 | Melanoma | 0.024906943 |
| mmu05215 | Prostate cancer | 0.026338425 |
| mmu05210 | Colorectal cancer | 0.027710529 |
| mmu05140 | Leishmaniasis | 0.027710529 |
| mmu04010 | MAPK signaling pathway | 0.029953682 |
| mmu05214 | Glioma | 0.030730368 |
| mmu04612 | Antigen processing and presentation | 0.032414039 |
| mmu04145 | Phagosome | 0.035360247 |
| mmu04514 | Cell adhesion molecules (CAMs) | 0.035515591 |
| mmu04115 | p53 signaling pathway | 0.037460209 |
| mmu05169 | Epstein-Barr virus infection | 0.03772521 |
| mmu04014 | Ras signaling pathway | 0.040406858 |
| mmu05202 | Transcriptional misregulation in cancer | 0.042356464 |
| mmu05332 | Graft-versus-host disease | 0.042404358 |
| mmu04144 | Endocytosis | 0.047739792 |
